# An assessment of flood vulnerability and adaptation: A case study of Hamutsha-Muungamunwe village, Makhado municipality

**DOI:** 10.4102/jamba.v11i2.692

**Published:** 2019-06-24

**Authors:** Rendani B. Munyai, Agnes Musyoki, Nthaduleni S. Nethengwe

**Affiliations:** 1 Department of Geography and Geo-Information Sciences, University of Venda, Thohoyandou, South Africa

**Keywords:** Vulnerability, Flood Susceptibility, Exposure, Flood Vulnerability Index, Resilience and Adaptation

## Abstract

This study assesses flood vulnerability, levels of vulnerability, determinants of flood vulnerability and coping strategies for flood hazards. The vulnerability and resilience of the local communities are key concepts in this study. Most households are vulnerable to flood hazards. It is therefore important to measure their levels of vulnerability and assess their responses for current and future planning. A flood vulnerability index was used to measure the extent of flood vulnerability. Key informant interviews, field surveys and household questionnaires were used to collect the data. The results show that vulnerability to flood in this community is determined by the nature of soil, dwelling type, employment, education and amount of rainfall in a season. Social and economic components scored higher than the physical environment, while social factors are higher than the economic factors. Contextual coping strategies in this community were temporary relocation, evacuation to a safe area and waiting for government and neighbours to help. The study recommends that public awareness campaigns, early warning systems and improved disaster management strategies must take into consideration differentiated levels of vulnerability and community coping mechanisms and preferences.

## Introduction

Floods are among the most devastating natural hazards and cost many lives every year (Dilley et al. 2005:43). To reduce the damage of floods, both structural measures, such as the building of dams and dikes, and non-structural measures, such as forecasting and education, are often employed (Jelmer 2013:1). Drogue et al. (2004:355) postulated that the frequency of floods has been rising every year. An increase in the frequency of floods resulted in loss of people’s lives, damage to property and infrastructure, as well as the destruction of the natural environment.

The number of people at risk has been growing each year and the majority are in developing countries with high poverty levels, making them more vulnerable to disasters (UN/ISDR 2004:95). However, communities and societies have specific ways of responding to floods, which has resulted in various ways of coping with the flood phenomenon. Cardona (2003:7) noted that individuals and communities are differently exposed and are vulnerable to floods because of the socio-economic factors, such as wealth, education, race, ethnicity, religion, gender, age, class, disability and health status. This is because flood vulnerability and adaptations are firmly related to the context of the natural environment and socio-economic factors of a specific area. The assessment of both vulnerability and adaptation are of great importance globally.

In South Africa, the annual risk of flooding is 83.3% and the level of vulnerability is high because of economic factors and geographical location (Zuma et al. 2012:127). According to Prevention Web (PRW 2011), ‘[i]n Eastern Cape, KwaZulu-Natal, the North-West and Limpopo provinces of South Africa; 77 flood disaster events were recorded in between 1980 and 2010’. This means that of all natural hazards, floods are the most frequently experienced disaster. Losses have been experienced by various communities around the world because of floods. Flood hazard is not only a local or regional issue but also a global issue, which should be planned and prepared for at international, national, provincial and local levels. Every year the World Health Organization (WHO) records various events of flood disasters in various regions of the world.

Floods can be caused by the natural environment through heavy rainfall, storms and cyclones, yet human activities such as development and settlement planning are key attributes. Nevertheless, climate change has become one of the important causes of floods, as it is believed that the rise in global temperatures will result in severe floods in several regions of the world. These changes are associated with the variability of weather and climate, such as the El-Nino Southern Oscillation (ENSO).

The aim of this study was to assess flood vulnerability and adaptation strategies of the Hamutsha-Muungamunwe rural community. To achieve the objective, the study set out to answer the following research questions: ‘what are the contextual determinants of flood vulnerability?’, ‘what is the extent of flood vulnerability?’ and ‘how do communities cope with floods in Hamutsha-Muungamunwe village?’

**FIGURE 1:**
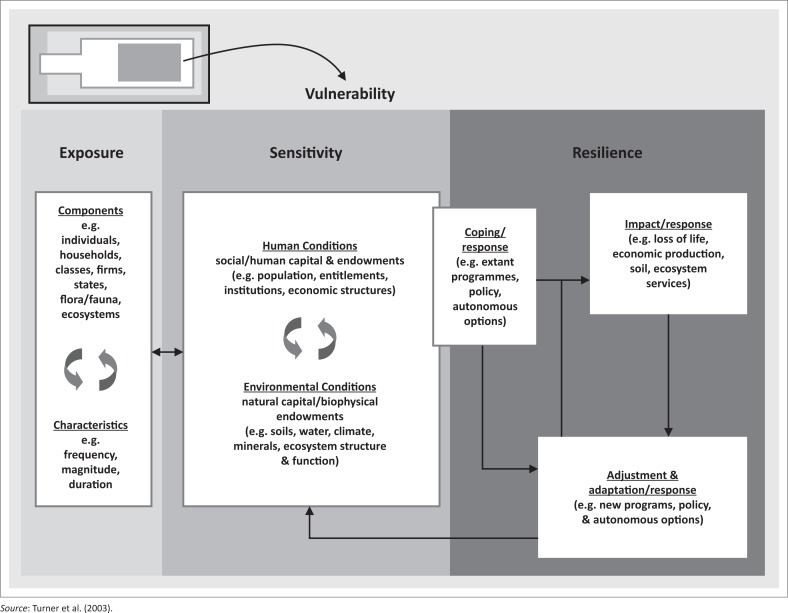
The conceptual framework of Turner et al.

This study adopted the conceptual framework of Turner et al. (2003:3), which illustrates that vulnerability is a function of exposure, sensitivity and adaptive capacity (see Figure 1).

Exposure, sensitivity and resilience are the key factors of vulnerability. Exposure refers to the alteration of the operational system, operating out of its normality operation. Judy et al. (2011:6) stated that it is the state and change in external stresses that a system is exposed to. The system is now predisposed to harm; these are also the present natural conditions and societal aspects.

Susceptibility is the potential or the likelihood of a hazard to have impacts in the system. According to Samuels et al. (2009:1), susceptibility is the probability of negative consequences of floods to the environment and society. Both socio-economic and the natural environments might be susceptible to a hazard. Resilience is the capacity of a community to adapt to changes in a hazardous area by modifying itself to achieve an acceptable structural and functional level (Galderisi et al. 2005). This means that the system must bounce back after disturbances, that is, the ability to retain the operation and function of the system is resilient.

According to the Intergovernmental Panel on Climate Change (IPCC 2007:881), ‘[S]ensitivity refers to the degree to which a system is affected, adversely or beneficially, by a given exposure’. Socio-economic and environmental systems have different sensitivities. Sensitiveness is mostly concerned with impacts of floods. A system can be sensitive to direct (physical) impacts (e.g. a given change in rainfall which affects the water supply of a city) and indirect (socio-economic) impacts (e.g. age structure of a population which influences the degree to which mortality increases during a heatwave) (Judy et al. 2011:6).

Vulnerability is therefore the degree to which a system is susceptible and unable to cope with adverse effects of climate change (IPCC 2007:21). Adaptation is the adjustment in natural or human systems in response to actual or expected climatic stimuli or their effects, which moderates harm or exploits beneficial opportunities (IPCC 2007:867). Literally, the definition of flood vulnerability is firmly rooted in how people or societies are likely to be affected by flood phenomena – that is, the sensitivity of the community or people to flooding considering the socio-economic, environmental and physical components. These components can be understood through an assessment of flood vulnerability components and factors.

Flood vulnerability is influenced by personal or group characteristics in terms of their capacity to anticipate and cope with the impacts of flood (Scoones 1998:8). Vulnerability quantifies the associated risks within the context of environmental and socio-economic capacity to adapt to flood events. Different social groups or classes within a society are differentially at risk, both in terms of probability of occurrence of an extreme flood event and helping different classes to recover (Cardona 2003; Nethengwe 2007:2). Ngie (2012:52) postulates that for vulnerability to exist, the capacity of the population to absorb, respond and recover from the impacts must be taken into consideration. This study therefore assessed the vulnerability of a community in a rural setting – Hamutsha-Muungamunwe community in the Vhembe district of the Limpopo province.

## Methods and materials

Qualitative and quantitative research designs were implemented in this study. Qualitative methods seek to better understand respondents’ own perceptions of vulnerability and capacities to cope with and adapt to possible threatening climatic events, as opposed to quantitative modes of inquiry (Jean-Baptiste et al. 2010:48). In quantitative research, the design is more deterministic in methodological approaches with fixed basics, determining what strategy or design the research should implement.

The target population included community leaders and members of the Vhembe District Disaster Risk Management Centre who were purposively selected. The total number of households was 810, and 60 were sampled through systematic random sampling and questionnaires were administered. Qualitative key informant interviews, questionnaires and field observations were used to collect the data. The key informant interviews were held with two community leaders and one member of the Disaster Management Centre. Babbie and Mouton (2001:476) postulated that the basic objective of a questionnaire is to obtain facts and opinions about a phenomenon from people who are informed on a specific issue. The questionnaire covered flood vulnerability determinants; indicators of flood vulnerability; impacts of flood on socio-economic status such as education, agriculture, health, infrastructure, housing and properties and water. Census (2011) provided useful information on flood vulnerability indicators such as population density, total population, types of sanitation and dwelling types. Meanwhile, the South African Weather Service (SAWS) provided rainfall data.

Data were entered into an Statistical Package for the Social Sciences (SPSS) spreadsheet wherein cross-tabulation was performed to interlink various variables in order to deduce any relationships between them. Descriptive statistics were applied (mostly frequencies) to enable the comparison of results either in percentages or in frequencies. The data were then grouped and presented in the form of tables, charts (bar or pie) and bar graphs.

A flood vulnerability index (FVI) was applied to measure the extent of flood vulnerability. The FVI method uses three factors of flood vulnerability, namely, exposure (E), susceptibility (S) and resilience (R). Exposure and susceptibility positively influence vulnerability, whereas resilience negatively influences vulnerability. Because of exposure, susceptibility and resilience have an influence on flood vulnerability. Indicators belonging to exposure and susceptibility increase the FVI; therefore, they are placed in the numerator. Indicators belonging to resilience decrease the FVI; therefore, they are placed in the denominator (Quang et al. 2012:103).

There are four major components of flood vulnerability, namely, social, economic, environmental and physical components. The three factors of vulnerability index are aligned with the components to reveal indicators. The general formula for FVI is calculated by classifying the components into three groups of indicators, namely, exposure, susceptibility and resilience (Balica et al. 2012:68). The formula for FVI is as follows:

FVI=(E×S)÷R or (E+S)−R [Eqn 1]

where E is exposure, S is susceptibility and R is resilience.

Pilot surveys, questionnaires, key informant interviews, Census 2011 and field observations were useful sources of indicators. The first aspect in the selection of an indicator was the provision of predetermined indicators, such as the level of income, type of house and questionnaires. Predetermined indicators were adopted from the study of Balica (2012:53). These indicators were then reviewed and merged to suit the study area. Table 1 presents the analysis and interpretation of the FVI. The index gives a number from 0 to 1, signifying low or high flood vulnerability.

**TABLE 1:** Interpretation of flood vulnerability index.

Index value	Description
Less than 0.1	Very small vulnerability to floods
0.01 to 0.25	Small vulnerability to floods
0.25 to 0.50	Vulnerability to floods
050 to 0.75	High vulnerability to floods
0.75 to 1	Very high vulnerability to floods

*Source:* Balica et al. (2012).

## Results

### Factors that determine flood vulnerability

**FIGURE 2:**
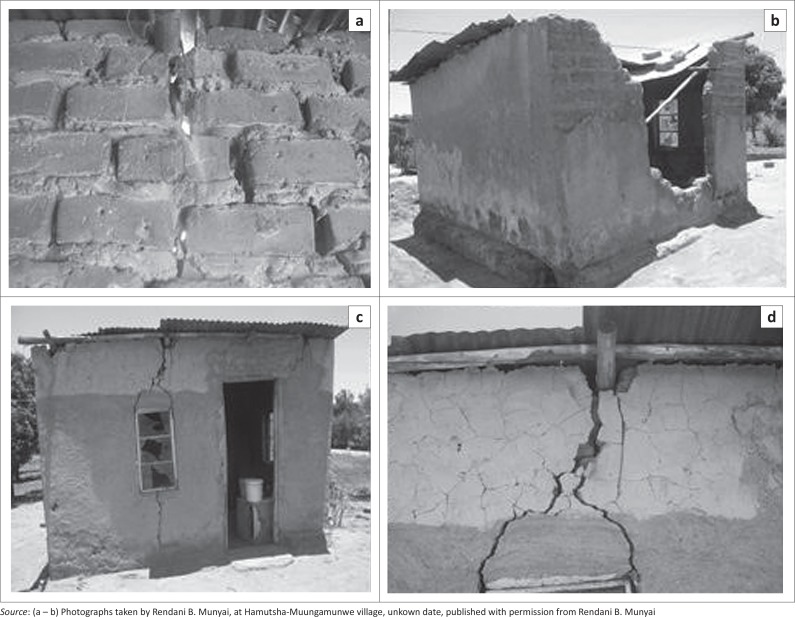
(b & c) Collapsed houses and (a & d) cracks in Hamutsha-Muungamunwe village.

Source: (a - b) Photographs taken by Rendani B. Munyai, at Hamutsha-Muungamunwe village, unkown date, published with permission from Rendani B. Munyai

Five factors that determine flood vulnerability are identified in Hamutsha-Muungamunwe village. These include the nature of soil, dwelling type, employment status, education and rainfall. All these factors were ranked according to their importance by using a five-point ranking scale, from most important to second, third, fourth and least important. The nature of the soil was ranked as the most important factor, followed by dwelling types. Respondents’ rankings were influenced by the collapsed and cracking of their houses that occurred during the flood event. The majority of respondents have experienced house collapse (see Figure 2). Employment status was ranked third, while education was ranked fourth and rainfall was ranked as the least important factor in determining flood vulnerability.

### The extent of flood vulnerability

Flood vulnerability index was applied to the Hamutsha-Muungamunwe community to measure the extent of their vulnerability to floods. Table 2 shows all indicators selected, components, factors of flood vulnerability, function and relationship with flood vulnerability. Fourteen indicators were identified from household level; the researcher used a deductive approach adopted from Balica (2012:58) and Jelmer (2013:8).

$${\text{FVI}}_{Social}=(\text{A}/\text{P},\text{ED}+\text{PD},\text{CH})-(\text{EM},\text{WS})$$ [Eqn 2]

where A/P: awareness/preparedness; ED: education level/literacy; PD: population density; CH: cultural heritage; EM: emergency service; and WS: warning system.

$${\text{FVI}}_{Economic}=(\text{UM},\text{QD}/\text{I}+\text{LU})-(\text{FI},\text{DSC})$$ [Eqn 3]

where UM: unemployment rate; QDI: quality of dwellings or infrastructure; LU: land use; FI: flood insurance; DSC: dam and storage capacity.

$${\text{FVI}}_{Physical\;environment}=(\text{FO}+\text{T},\text{HR})-(\text{DSC})$$ [Eqn 4]

where FO: frequency of flood occurrence; T: topography; HR: heavy rainfall; and DSC: dam and storage capacity.

**TABLE 2:** Vulnerability indicators, components, factors and relationship with vulnerability.

Indicators	Components	Factors	Function/relationship with vulnerability
Awareness/preparedness	Social	Susceptibility	Higher number of people aware, lower vulnerability
Education/literacy level	Social	Susceptibility	Higher number of people uneducated, higher vulnerability
Unemployment rate	Economic	Susceptibility	Higher %, higher vulnerability
Infrastructure/dwelling quality	Economic	Susceptibility	Higher % of good-quality dwelling, lower vulnerability
Frequency of flood occurrence	Physical/environmental	Susceptibility	Higher number of occurrences/year, higher vulnerability
Population density	Social	Exposure	Higher number of people, higher vulnerability
Cultural heritage	Social	Exposure	Higher number of CH, higher vulnerability
Land use	Economic	Exposure	Higher %, lower vulnerability
Topography	Physical/Environmental	Exposure	The steeper the slope, higher vulnerability
Heavy rainfall	Physical/Environmental	Exposure	Many days of heavy rainfall, higher vulnerability
Warning system	Social	Resilience	Having WS reduces the vulnerability
Emergency service	Social	Resilience	Bigger number of people, less vulnerability they are
Flood insurance	Economic	Resilience	Higher number of FI, lower vulnerability
Dam and storage capacity	Economic and physical /environmental	Resilience	Higher capacity, lower vulnerability

CH, cultural heritage; WS, warning system; FI, flood insurance.

The FVI results of Hamutsha-Muungamunwe village are presented in Table 3. Social and economic components scored higher vulnerability to floods than the physical environment, whereas social factors are specifically higher than economic factors in terms of vulnerability. This means that socially Hamutsha-Muungamunwe community has a very high vulnerability to floods. This is because of the high population density, lack of early warning systems for flood and poor or slow emergency services. Economically, Hamutsha-Muungamunwe community has a ‘high to very high’ vulnerability to floods. The physical environment contributes the least, with ‘small vulnerability to floods’ because of fewer days of heavy precipitation and plain landscape.

**TABLE 3:** Hamutsha-Muungamunwe flood vulnerability index results.

FVI components	FVI values	FVI designation
FVI Social	0.801	Very high vulnerability to floods
FVI Economic	0.752	High vulnerability to floods
FVI Physical Environmental	0.183	Low vulnerability to floods
FVI Total	0.578	High vulnerability to floods

FVI, flood vulnerability index.

### Coping strategies

**FIGURE 3:**
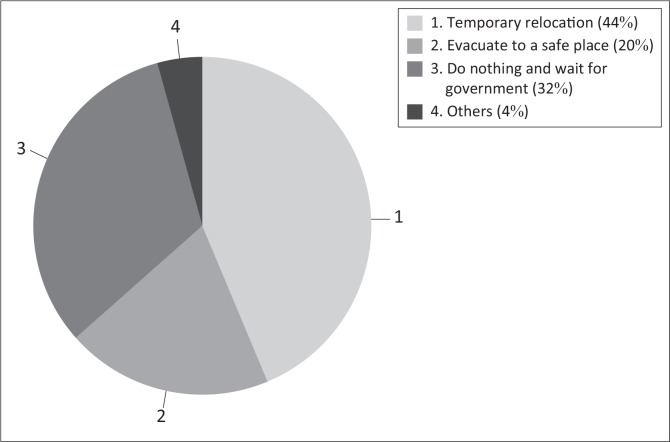
Coping strategies adopted by the community in Hamutsha-Muungamunwe.

Considering all impacts of floods, all coping strategies were assessed. Figure 3 presents the coping strategies implemented against floods. The most preferred coping strategy was temporarily relocating, as confirmed by 44% of respondents. The second coping strategy was relying on neighbours and government help to withstand flood impacts, as indicated by 32% of respondents. The third strategy was evacuation, which was stated by 20% of the respondents. The remaining 4% of respondents stated other strategies, including making a small furrow to redirect flood water and planting lawn grass in and around the constructed houses.

## Discussion

In this section, we discuss the flood vulnerability determinants, namely, nature of the soil, dwelling type, employment status, education and amount of rainfall. These determinants do not function independently but are interconnected with each other. Pelling (1997:202) found that income level, dwelling type, health and resource accessibility are factors that determined flood vulnerability in Georgetown, Guyana. However, the result of this study is different because instead of income level, health and resources accessibility as the determinants of flood vulnerability, the nature of soil, employment, education and rainfall were apparent. The only similarity is the presence of dwelling type; however, it was ranked as the second important factor determining flood vulnerability in this community.

The nature of soil is identified as the most important factor determining flood vulnerability in Hamutsha-Muungamunwe village. This is because of the dominance of clayey soils in this area. The topsoil is composed of sand, while the bottom part is clay and can hold water preventing any permeability process to take place. The exposure to flood is also directly related with flood flow (Karmaka et al. 2010:129). Clayey soil influences the probability of flood occurrence and vulnerability of people to floods. More permeable soil has more infiltration capacity and therefore reduces surface run-off, whereas less permeable soil has less infiltration capacity and is more prone to water logging (Grosshans et al. 2005:40). The findings of this study revealed similarities with the study conducted by Karmaka et al. (2010:129), where the nature of the soil was found as one of the major factors determining vulnerability to floods. A small downpour of rain can cause floods because of the nature of soil in this area.

Studies conducted by Balica (2012), Jelmer (2013) and Samuels et al. (2009) showed that rainfall and education level rankings are among the most important factors that determine flood vulnerability; however, in this study, they are found the least. This proves that vulnerability is rooted within the context of the socio-economic characteristics and the physical environment. The study of flood vulnerability should not be generalised because findings of a certain area might not be relevant to another area. The Hamutsha-Muungamunwe community should be aware of the nature of soils in their area and this should always be considered always in any construction and settlement processes. It also applies to the type of dwelling that should be built in this area because a poor type of dwelling increases the vulnerability to hazards.

Social factors influenced the vulnerability to floods more than economic and physical environments. The total vulnerability of the study community is within the parameter of ‘high vulnerability to floods’. Even though social factors increase vulnerability than both economic and physical environments, there is sufficient evidence to recognise the contribution of economic factors in their vulnerability because the value of FVI social is 0.801, while the value of FVI economy is 0.752. This means that socio-economically, this area is highly vulnerable to floods.

However, the incorporation of flood vulnerability designations is probably the most difficult of all variables to include in the vulnerability index (Balica et al. 2012:45). A very high vulnerability to floods is associated with high extreme potential for loss in both the socio-economic and physical environments. This kind of vulnerability can also result in catastrophic phenomenon. High vulnerability indicates that there is high potential for damage to properties and loss of life, and this is normally based on the indicator of the lack of flood warning system. High vulnerability is assigned to a case where there is a high chance for loss of life, while medium vulnerability is allocated in a case where medium potential of harm to people’s lives and properties is apparent. A small vulnerability is assigned if there is only small potential of harm and damage to the socio-economy of a place, while very small vulnerability is concerned with a very small potential damage and harm upon various systems within a particular place. These losses and damages occur in both the socio-economy and the physical environment. The existence of quantifiable data is important for flood vulnerability measurement.

The high vulnerability of Hamutsha-Muungamunwe village is closely associated with the community’s capacity to cope with floods; however, poverty also plays a significant role.

From the above discussion, it is clear that vulnerability is not only a physical condition but also that people are significantly exposed to hazards socially. The most critical part about these socio-economic characteristics is that they are very contextual (Balica 2012:68). Vulnerability of a certain place cannot be generalised to other settings; otherwise, the results will be misleading.

Balica et al. (2012:14) found that social and economic components are more significant than the physical environmental component, which is similar to the findings of our study. This reveals that levels of social and economic vulnerability are important because of the ability of these factors in assisting people to resist and return to their normal state of operations. Stronger socio-economic characteristics of a specific area influence better resilience to floods. However, the similarities between the findings of these two studies do not mean that every FVI would result in higher social and economic indices than the physical environmental factor. This is because flood vulnerability is rooted within the parameter of scale and time, and it is dynamic to change in space, time and place (Balica et al. 2012:74).

The main problem associated with these indicators as listed in the literature is the availability of the data. Groundwater level was not included in this study, although significant, because of their unavailability. Any future studies would have to take them into consideration. This study found that exposure, susceptibility and resilience influence flood vulnerability in the study area.

### Coping strategies

Various coping strategies were assessed based on the respondents’ capability of living and resisting floods. Socio-economic characteristics of the respondents play a key role in determining their ability and capacity to respond during flood events. Various studies have identified different coping strategies; for example, Ngie (2012:62) found that relocating to a safer area and evacuation were the most practised coping strategies in the study that was conducted in Diepsloot. The present study has similarities with Ngie’s finding because it revealed that evacuation and relocation are part of the coping strategies in Hamutsha-Muungamunwe community. In addition to similar findings, ‘waiting for government and neighbours to help’ was also part of the coping strategies practised in this community.

High rate of unemployment and low income among the respondents have contributed to the lack of resilience of Hamutsha-Muungamunwe community. This is because resource availability influences the level of resilience and recovery. Adequate resources lead to lower vulnerability, whilst a lack of them makes people more exposed and vulnerable to floods because it would take a long period for people to recover from damage. Temporary relocation was the most adopted coping strategy because of good communication and interconnection between neighbours and relatives. This coping strategy is rooted within the parameter of relationships between people, meaning that where there is a lack of communication and interconnection, this coping strategy is not possible. A person cannot temporarily relocate to a neighbour’s or relative’s house without any good relations with them.

However, some respondents prefer to wait for the government and neighbours to help. This is unadvisable because the municipality has insufficient resources for recovery and responses during flood events. Sometimes they are unable to access relief funds because of a lack of capacity for assessing flood impacts to lodge a declaration of disaster with the National Centre for Disaster Management. The majority of the respondents therefore did not receive any help from the municipality. With the very high vulnerability to floods by the community, there is a need to develop a strong resilience system to meet these high levels of vulnerability to flood.

Tsi-Hamutsha, a community organisation, helps in recovery and response. The critical point is that few respondents have interacted with this organisation. Most of the members rely on neighbours and relatives for relocation when their houses collapsed and inform the community leader. Even though the organisation is not made specifically for flood support, it is quite helpful because most of the respondents are not aware of other disaster risk management options available to them.

## Conclusion and recommendation

The main objective of this article was to assess flood vulnerability and adaptation strategies of Hamutsha-Muungamunwe village. The findings articulated the following factors that determine flood vulnerability, in order of importance: soil nature, dwelling type, employment status, education and amount of rainfall. The present socio-economic characteristics of Hamutsha-Muungamunwe village have influenced the vulnerability of this area. The findings also revealed that this area is socially, economically and environmentally vulnerable to floods at different levels. Meanwhile, the physical and environmental components have minor contribution in the vulnerability of this area to floods. The overall finding of Hamutsha-Muungamunwe village’s vulnerability to flood indicated that the area has high vulnerability to floods. The main reason of this high vulnerability to floods is the lack of resilience.

There are many coping strategies that the community is using and intends to use against floods. Of all the identified strategies, temporary relocation to safer places such as houses of relatives and neighbours, doing nothing and waiting for neighbours and government to help and evacuating to a safer area were among the most practised coping strategies.

To build resilience against floods, the following initiatives are recommended:

- There is a need for public awareness campaigns, particularly the development of efficient early warning systems. The availability of an early flood warning system will assist households in the expansion of their knowledge. Indigenous knowledge systems should be taken into consideration when developing these systems.
- The community needs to develop strong collaboration with traditional leaders, municipal officials, Makhado municipality, Vhembe district and households.
- The municipality should commit more funds for disaster reduction activities.
- It is important to build dwellings with durable materials, which requires channelling of extra funding to the community for housing.
- Community leaders should not allocate plots in the floodplain area. Hence, there is a need for floodplain mapping.
- The community leaders should develop response and recovery mechanisms by setting aside money for flood response and recovery.
